# Towards a collaborative, global infrastructure for biodiversity assessment

**DOI:** 10.1111/j.1461-0248.2007.01063.x

**Published:** 2007-08

**Authors:** Robert P Guralnick, Andrew W Hill, Meredith Lane

**Affiliations:** 1 Department of Ecology and Evolutionary Biology, University of Colorado at Boulder Boulder, CO 80309, USA; 2 University of Colorado Museum, University of Colorado at Boulder Boulder, CO 80309, USA; 3 Global Biodiversity Information Facility Secretariat, Universitetsparken 15 2100 Copenhagen, Denmark

**Keywords:** BioGeomancer, data visualization, Geographic Information Systems, Global Biodiversity Information Facility, global biodiversity services, Google Earth, species richness estimation, survey gap analysis

## Abstract

Biodiversity data are rapidly becoming available over the Internet in common formats that promote sharing and exchange. Currently, these data are somewhat problematic, primarily with regard to geographic and taxonomic accuracy, for use in ecological research, natural resources management and conservation decision-making. However, web-based georeferencing tools that utilize best practices and gazetteer databases can be employed to improve geographic data. Taxonomic data quality can be improved through web-enabled valid taxon names databases and services, as well as more efficient mechanisms to return systematic research results and taxonomic misidentification rates back to the biodiversity community. Both of these are under construction. A separate but related challenge will be developing web-based visualization and analysis tools for tracking biodiversity change. Our aim was to discuss how such tools, combined with data of enhanced quality, will help transform today's portals to raw biodiversity data into nexuses of collaborative creation and sharing of biodiversity knowledge.

## Introduction

### The need for Internet-based biodiversity data and tools

The planet is in the midst of a biotic crisis almost certainly caused by human activities (Wilson 1988; Heywood & Watson 1995; Sala *et al.* 2000; Hoekstra *et al.* 2005; Loreau *et al.* 2006). This crisis is of an unprecedented scope and rate, and may lead to half the species on earth going extinct by the end of this century (Pimm *et al.* 1995; Jenkins 2003). The ability to track the predicted continued changes to the diversity and distribution of Earth's organisms in relation to environmental factors is a key tool for defining strategies and mechanisms for conserving our current biodiversity (Niemel 2000). Such ability is also essential for understanding and predicting future responses of biodiversity to shifting landscapes and changing climate.

A major impediment to advancing our biodiversity knowledge is the paucity of digital species-occurrence data available online. Although more species-occurrence records are steadily being acquired, it is still difficult to find past and current biodiversity data for anything but well-studied taxa that occur in well-studied areas. It has been even harder to aggregate data from multiple sources in order to ask new questions not envisioned by those performing the initial surveys. We are often unable to answer very simple, fundamental biodiversity questions for most regions in the world, such as ‘What biodiversity has been found in region X?’ and ‘Has previous sampling been sufficient to support confidence in biodiversity estimates?’

### Initial approach to global biodiversity occurrence data availability

A partial solution to the problem of data availability is a global mechanism that facilitates sharing of biodiversity data that is housed in natural history collections throughout the world. These collections hold vast troves of specimens collected over three centuries. This legacy includes on the order of 1–2 billion records of biological diversity (Beaman & Conn 2003), but only a small proportion (*c.* 5–10%; Krishtalka & Humphrey 2000) are digitized. It is especially important to note that a substantial percentage of the total are records collected prior to major alterations of native landscapes. Thus, these specimen data are the best possible resource with which to construct baselines to measure changes in biodiversity over time (Graham *et al.* 2004; Suarez & Tsutsui 2004), though collection institutions often lack the funding to digitize their specimen data.

The Global Biodiversity Information Facility (GBIF) has developed a worldwide information infrastructure through which natural history collections (as well as other institutions and organizations) can publish their databases, and thus become part of a distributed global network of shared biodiversity data (Edwards 2004; Lane 2006). Any user with Internet connectivity can access a vast queryable global biodiversity data service. As of April 2007, the GBIF data portal mediates access to *c*. 120 million species-occurrence records from over 1000 collections housed in *c*. 200 institutions in *c*. 34 countries. Because all data adhere to a common set of standards for data and metadata (Graham *et al.* 2004) and use the same methods for sending data over the Internet (Stein & Wieczorek 2004), GBIF search results are returned to the user in a common format.

### Current limitations to effective use of global biodiversity data

Despite the quantum leap forward in the development of mechanisms for aggregating species-occurrence data that the GBIF data portal represents, much more will need to be done to make the portal function effectively as part of a global infrastructure for biodiversity assessment. Limitations of the system as it stands fall into three categories: (i) quality and utility of the taxonomic and geographic data associated with species-occurrence records; (ii) low levels of sophistication of search mechanisms for acquiring species-occurrence records and (iii) the difficulty of linking raw species-occurrence data with existing visualization and analysis tools that encourage collaboration and the same time enhance workflows in biodiversity science.

The first problem with the existing GBIF portal is that simply making species-occurrence records available is not sufficient to assure their use by the community, especially if the records are considered untrustworthy by potential end-users. The minimum requirements for a species-occurrence record are its taxonomic identification, together with when and where the specimen was collected. All three data types are prone to errors (Chapman 2005a, b) though in this paper we do not address errors in collection dates. Thus, it is important to have methods for assessing the amount and types of errors in these fundamental data types.

There are two main types of taxonomic identification errors. The first occurs when a specimen is labelled with a name that is outdated or otherwise invalid. The second and more vexing type of error arises because in taxonomically difficult groups of organisms, misidentifications at the species level are common. Rates of specimen misidentification range from 5% to as high as 60%, and thus use of such data would be misleading (Meier & Dikow 2004). Mechanisms are therefore needed for reporting (i) the degree of confidence that may be placed in identifications and (ii) new taxonomic and systematics findings in such a way that specimen identifications can be updated to reflect the current state of taxonomic knowledge.

Another problem with legacy data is that although geographic locality information is nearly always present in the form of a textual locality description, it is often not in a form suitable either for computer mapping or assessment of accuracy. To be computer-mappable, these verbal descriptions need to be converted into latitude and longitude coordinates through a process called retrospective georeferencing. This mappable representation includes not only the geospatial coordinates, but also a measure of ‘uncertainty’ around those coordinates. Coordinates with high uncertainty are likely to be unsuitable for biodiversity analyses at fine spatial scales, although they may have utility at scales with lower degrees of granularity.

The retrospective georeferencing process is an immense undertaking because only a fraction of the legacy data from natural history repositories are either already georeferenced or have associated Global Positioning Systems coordinates. Worse, because best practices for georeferencing have only recently been developed (Chapman & Wieczorek 2006), existing retrospective georeferences are often of low accuracy, unstandardized and/or undocumented (Guralnick *et al.* 2006). These factors make it difficult to combine data because it may not be possible to compare the quality of georeferences.

Along with data quality concerns, there are also issues with the current implementation of global biodiversity data services. One problem is that the prototype GBIF data portal allows selection of a geographic area of interest at the level of country. For example, one cannot ask ‘What mammal species have been found on Bering Island?’ or ‘What bird species are located in pinyon pine forests in the Rocky Mountains of North America?’ Limitations of this sort decrease the likelihood that resource managers and conservation planners, who are typically more interested in ecologically defined areas, will utilize these systems. However, a new GBIF data portal with increased functionalities, including the ability to search by user-defined geographic bounding boxes, is in preparation for launch in July 2007.

Perhaps the greatest impediment to developing a collaborative, global infrastructure for biodiversity assessment has been the disconnection between the raw data available from sources like the GBIF data portal, and data visualization and analysis tools. Growth in networked biodiversity data content has been matched by growth and refinement of such tools, but these events have happened largely independently.

Three particular methodological areas of interest in the community have been ecological niche modelling (Peterson 2001; Soberón & Peterson 2004), species richness estimates (Soberón *et al.* 2000; Ponder *et al.* 2001; Rahbek & Graves 2001; Petersen *et al.* 2003; Meier & Dikow 2004; Guralnick & Van Cleve 2005) and tools for survey development (Funk *et al.* 2005). These approaches complement spatial ecological analyses like those packaged in the program spatial analysis in macroecology (http://www.ecoevol.ufg.br/sam/; Rangel *et al.* 2006). Other available desktop and online software (e.g. desktopgarp, http://nhm.ku.edu/desktopgarp/; estimates, http://purl.oclc.org/estimates; eco-tools, http://www.eco-tools.net) provide the logic and some of the geographic and environmental data for generating results. However, there is currently no piece of software that provides a means to effortlessly accumulate and explore the existing, up-to-date, georeferenced biodiversity data that is now available from computer networks.

Nonetheless, there is great potential for a new approach to acquiring and sharing information and knowledge about biodiversity because of this parallel development of data portals and analysis tools, together with increased Internet speed. Scientists can use existing workflows (or create their own) that link data from portals with web-enabled visualization and analysis tools to streamline the process of performing biodiversity science (Guralnick & Neufeld 2005). Doing so will hasten generation of new biodiversity knowledge and sharing it with the widest possible audience. We elaborate this vision in more detail below.

## Methods for increasing biodiversity data quality

### Taxonomic name services

The as-yet unrealized, ideal solution for taxonomic searching is to cross-reference searches against an online database compiled by taxonomic experts that contains all known species names as well as associated synonyms. Fortunately, there is ongoing development of web-based taxonomic name database services. The most nearly complete authoritative taxon name database is the Catalogue of Life (CoL), a joint project of the Species 2000 and Integrated Taxonomic Information System (ITIS) partnership (Bisby *et al.* 2005). This collaborative database currently contains names for *c*. 57% of all named biological species, and it is expected to grow to 95% of all known species by 2011. To date, the GBIF portal has utilized the CoL as the core of its Electronic Catalogue of Names of Known Organisms (ECAT) and for navigation of its taxonomic browser. In the new portal implementation, GBIF will continue to utilize CoL, but ECAT will also incorporate additional databases as well as algorithms that will allow users to choose among several possible classifications, and return the species-occurrence records of interest under the set of taxon names valid in that classification as well as their synonyms.

### Taxonomic data quality assessment and validation

The more difficult problem of misidentified taxa is being addressed by two linked endeavours that will help to mitigate the problem. First, digitization and automated markup of all taxonomic literature to delineate important taxonomic, anatomical, locality and other information in original descriptions and revisions of a taxon have been proven in concept (Koning *et al.* 2005). The major natural history museum libraries are collaborating to digitize their works through the Biodiversity Heritage Library Project (http://www.bhl.si.edu). Once this process is underway, species-occurrence records in biodiversity data portals can be linked to taxon names in the literature so that data stewards and users can better estimate specimen identification accuracy.

Second, an important but still nascent next step will be to improve dissemination of corrected identifications. Taxonomic experts often check and correct misidentifications in collections, but traditional practice has not been conducive to reporting these corrections to the community at large. This is the reason that, ideally, GBIF data providers themselves maintain the occurrence data that they share with the network, and as needed update the names applied to those records with an annotation as to the recency of update. The taxonomic community also acknowledges that some groups are more taxonomically difficult than others. This is metadata that should be linked to species-occurrence records so that potentially naive end-users would be able to determine which groups have well vetted taxonomies and highly accurate specimen identifications. This solution is preferable to withholding data for problematic groups, because identifications at a more inclusive taxonomic rank (e.g. genus or family) may be accurate, even though included species-level identification(s) is/are not.

### Retrospective georeferencing

A major development in georeferencing that is likely to have strongly positive impacts on both the quality and efficiency of the georeferencing task is the international, collaborative BioGeomancer project, which has led to two main products. The first is a best practices (as agreed upon by the experts in the field) guide to georeferencing (Chapman & Wieczorek 2006). The second, which encodes these best practices, is a semi-automated and web-based workbench for performing georeferencing tasks (Guralnick *et al.* 2006). The workbench allows any user in the world to submit locality descriptions, and retrieve and edit both geographical coordinates and an automatic calculation of uncertainty for each locality. The BioGeomancer workbench will greatly increase the speed of retrospective georeferencing of data records as they are made available via the GBIF portal, and increase the accuracy and comparability of the results. The workbench and more details are available at http://www.biogeomancer.org

## Suggestions for increasing the utility of the GBIF data portal

### Visualizing biodiversity data

So far, we have largely focused on how the breadth, accuracy and reliability of data available from an open and freely available network of primary data providers can be improved. Yet, fundamental questions remain: ‘How can high quality information from a global biodiversity portal be used to support research, conservation management and education?’ and, ‘How can ecological informaticians build tools that put the GBIF data portal to use to facilitate those goals?’ The biodiversity community needs to effectively and quickly mobilize data and tools to create knowledge that can be shared with the larger scientific community, policy-makers and the general public, given the current biodiversity crisis (Wilson 1988).

To do this, we must develop a web-based system that links biodiversity data from services such as GBIF with tools that allow users to generate and share knowledge about biodiversity. In the short-term, the major function of emerging global biodiversity informatics services will be to provide a first-pass view of biodiversity at regional to continental scales. Therefore, the first step will be to build visualization and analysis services to answer fundamental questions such as: ‘How much existing biodiversity data is there for a region of interest and is it enough to determine accurate measures of species richness?’ or ‘Where is the most likely spot to survey for more biodiversity given the current distribution of samples and current environmental conditions?’ At the same time, we also need to develop solutions that contribute to the longer term goal of building a collaborative, global infrastructure for biodiversity assessment.

**Figure 1:**
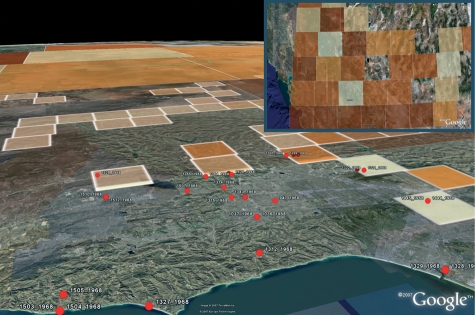
A screenshot from the Keyhole Markup Language of Species-Occurrence Record Density (KSORD) tool. In the foreground are individual record distributions of *Thomomys bottae* (Botta's Pocket Gopher) in western North America; at further distances from the point-of-view are progressively larger boxes that summarize record density in the given area. Darker tones represent greater density of records. Inset: The same region of western North America as viewed from altitude.

Integrated visualization tools are a key feature of these developments. Initially, visualizations can be produced that provide users with nearly immediate graphical summaries of species-occurrence data sets, thus allowing them to determine if those data sets might fit a research or management need. We have developed a prototype of one such tool, which uses GBIF data to construct a spatio-temporal species-occurrence record density map in the Keyhole Markup Language (KML), native to Google Earth®. The KML of Species-Occurrence Record Density (KSORD) is a web-based script that allows users (or other applications) to load species-occurrence records and generate a visual representation of record density using Google Earth. As the user moves from a global or continental view to more local, fine-scale views, the grids of observed abundances of taxa also increase in granularity, until the user can see the individual points on the map (Fig. 1). These outputs also utilize the ‘date collected’ field stored in GBIF-mediated records so that users may examine the change in record density over time as well as space. The KSORD tool and example outputs are available at http://ksord.colorado.edu

Another geospatial tool that links GBIF data with analysis tools more effectively than the use of KML is an online ‘flatmap’ GIS, which is more akin to traditional Desktop GIS programs than it is to virtual globes. This tool allows users to move or delete points on the map, perform spatial queries to search for specific points, and to send and return raster data layers when a biodiversity analysis is performed. This online GIS serves four essential purposes related to streamlining the workflow of doing biodiversity analyses. First, it provides an intuitive way for users to retrieve species-occurrence data from the GBIF portal. The user select regions of interest by drawing areas on online maps, and then queries the portal for data records that occur within the chosen area(s). Second, it serves as a means for displaying and querying other environmental data layers in conjunction with biodiversity data. For example, users can view and retrieve climatological or satellite data together with species distribution records. Third, users can visually validate occurrence records prior to downloading a full data set or performing an analysis online – obvious outliers can be examined for easily corrected errors vs. disjunctions of real scientific interest. Finally, it provides the backbone for further biodiversity analyses by feeding data to other applications or services that return summary spatial results. We envision that the GBIF data portal's online GIS will support core functions that include selection of regions and taxa of interest, returned to the user as record data and visual displays (flat-map or virtual globe). Users could then validate records, checking for appropriate taxonomy and geographic outliers.

### Web services and automation of workflow in biodiversity science

**Figure 2:**
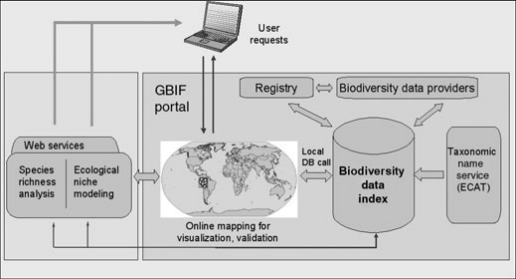
Diagram presenting an integrated workflow for biodiversity assessment. The Global Biodiversity Information Facility (GBIF) portal includes a biodiversity data index which caches a subset of the total incoming data from multiple providers. Two other GBIF portal services that are linked to this index are the taxonomic name service and an online GIS. The latter provides a means for users to view biodiversity in regions of interest and potentially modify and validate those data sets from which the viewed data are derived. Biodiversity analysis web services located anywhere on the Internet can ‘deep link’ to the GBIF portal such that data from the GBIF portal can be sent to these analytical services to perform for example, ecological niche modelling or species richness assessment. Results of the analyses that have employed GBIF-mediated data are returned to the user, with metadata that identify the source data provider(s), so that the analysis can be repeated and verified, and so the data provider(s) can be given attribution in any publication(s) resulting from the analysis(es).

Virtual globes and online GIS provide visual, as opposed to analytical, views of the data. In order to test hypotheses or make decisions, these visualization aids need to be linked not only to the GBIF portal, but also to analysis engines. Such linkages would provide end-to-end workflows for biodiversity research and management, similar to workflows in other ecological domains (Ellison *et al.* 2006). The next step in building capacity for such a workflow is to deploy software for methods such as ecological niche modelling or estimation of species richness as web services. Such services would receive data sets sent over the Internet by users or other applications, process the data and return summary results (Fig. 2). A user could then use this workflow to step through a full analysis from data acquisition through analysis to an ‘answer’ (presented via a visualization tool or statistical summary) to a biodiversity research, management or policy question.

**Figure 3:**
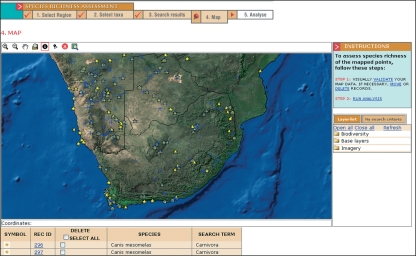
Screenshot of Global Biodiversity Information Facility (GBIF) Mapping and Analysis Portal Application at the map and validation step of performing a species richness analysis. In the previous step, a region of southern Africa was selected and searched for two mammal groups. The GBIF data cache returned 935 Carnivora records (yellow stars) and 5411 Rodentia records (blue stars), respectively. Using online GIS tools, users at this step can select occurrence data points, either on the map or in the table at the bottom of the web page, and move or delete records. Having performed this validation step, the user can then perform a set of analyses based on this occurrence data set.

The best current example of such an end-to-end, web-based workflow system for biodiversity analysis is the GBIF Mapping and Analysis Portal Application (GBIF MAPA). GBIF MAPA (http://gbifmapa.austmus.gov.au/mapa/) demonstrates how a combination of GBIF data, online GIS and analysis tools running as web services allow for biodiversity decision-making. Three analytical methods [environmental extraction analysis, species richness analysis, Survey Gap Analysis (SGA)] are available in GBIF MAPA. The initial workflow steps for each of the three are nearly identical until the final analytical step. Initially, users select a rectangular region of interest using a ‘rectangle-create’ tool available as part of an online GIS, and then taxa of interest. The application next allows the user to generate customized maps of the species-occurrence data in an online GIS (Fig. 3). User can select, move and delete records in the online GIS either by clicking on the map or by using a table of results displayed below the map. In the final step, the user performs a species richness analysis or SGA, or gets a table of environmental conditions at the spot where selected species occurrences were collected. Case studies showing how these tools can be used to answer ecologically meaningful questions are available in Flemons *et al.* 2007.

The species richness tool provides options for different grid sizes and then overlays the chosen grid on species occurrences in the region of interest. The summary of abundances of each species in each grid cell is sent to a web service that performs several different species richness estimation techniques. The user is then presented with a summary of incidence and abundance estimator coverages, number of singletons, doubletons, unique and duplicates, as well as randomized parametric and nonparametric analyses of species richness.

The SGA tool provides a means to design a biodiversity survey that will best complement existing survey records by identifying those areas least well surveyed in terms of environmental conditions (Faith & Walker 1996). It is also perhaps the best demonstrator of the power and potential of linking together GBIF data, online GIS and analysis engines. SGA operates by building a multidimensional environmental space for the region of interest and then determining how well the existing site data (in this case GBIF species-occurrence data) samples that space. It then creates a GIS layer classified into complementarity values. The areas with the highest such values are recommended as survey sites, because the SGA analysis maximizes the environmental representativeness of the survey effort. The tool returns this new GIS layer to the user, with the most complementary area ‘flagged’ with a map symbol. The user can adjust the symbol location if there is a nearby location that has, for example, better road or river access than the selected point. After determining the ‘best’ new survey site, the user can perform a new iteration of the analysis using the newly selected site in addition to the original sites. This process is reiterated until the user has generated many sites, at which point the spreadsheet of site locations can be downloaded.

### A case study in assessment of global biodiversity

In order to show how GBIF data may be used to provide an assessment of global biodiversity, we present a case study that replicates, across different parts of the world, a series of species richness experiments for two well-studied groups, birds and mammals. We chose to examine species richness because it is a fundamental parameter in conservation biology. We chose birds and mammals because taxonomic quality for these groups is high, they are well represented in the GBIF data cache, and they are often used in studies of global biodiversity hotspots (Ceballos & Ehrlich 2006).

**Figure 4:**
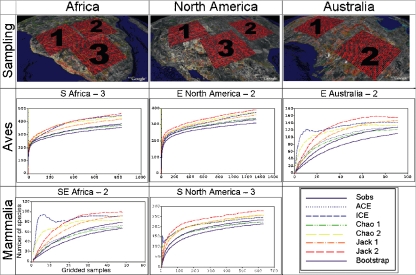
A Google Earth view of the sampling grids and resultant species richness estimates of birds and mammals for some of the nine equal area regions selected for comparison. The numbered grids for different areas are shown in the top row of panels. The middle and bottom rows show species richness estimate curves for birds and mammals, respectively. The numbering in each graph title refers to the numbered grid in the top panels. The graphs show that in some regions, estimators tend to give relatively similar estimates close to observed richness and nearly plateau (e.g. North America) while in other regions, data sets are not close to the maximum number of observable species given sampling methodologies (e.g. regions of Africa and Australia).

The approach to running these experiments differs in execution from how they would be conducted using the species richness tool in GBIF MAPA. We have instead built an automated ‘crawler’ that remotely queries GBIF for every avian and mammalian taxon documented in ECAT and accumulates the species-occurrence records for those taxa if they meet four criteria: the record must be more recent than 1950, identified to species rank, georeferenced with a coordinate precision of < 25 km or if lacking a precision value have an associated locality description, and located within one of nine predetermined sampling areas. The nine areas were selected to represent terrestrial regions across multiple continents (Fig. 4) that have likely been sampled at different intensities (e.g. higher in Europe and North America and lower in other areas). Each of the areas is 1000 × 1000 km and is divided into 1600 grid cells (25 × 25 km). Any record located within one of the nine areas was then recorded within the cell it occupied.

Records accumulated by the crawler were imported to estimates 8.0 (Colwell 2005) within which a suite of nonparametric species richness estimators were employed. We determined the mean number of observed species, and mean richness estimates using ACE, ICE, Chao1 and Chao2, Jackknife1, Jackknife2 and Bootstrap without replacement using 100 replicates. Table 1 shows summary data from the crawler including number of species in the sample, and ACE and ICE estimator results for bird and mammals in each of the nine areas. Figure 4 shows graphical estimator results for selected regions.

**Table 1:** Summary data for the Global Biodiversity Information Facility-based species richness assessment of birds and mammals across nine equally sized large regions on four continents

Location	Taxonomic class	Number of species	Number of individuals	Number of cells (1600 total)	ACE	ICE
W Australia	Mammalia	4	719	5	n/a	n/a
	Aves	5	13	5	n/a	n/a
E Australia	Mammalia	21	126	38	36.28	31.52
	Aves	110	570	90	137.32	141.74
Europe	Mammalia	66	16 725	136	71.66	89.49
	Aves	313	444 388	178	353.97	384.38
E North America	Mammalia	96	6718	384	107.51	123.63
	Aves	311	620 227	1335	341.04	374.83
W North America	Mammalia	132	7382	436	142.95	149.56
	Aves	290	205 437	757	315.54	340.93
S North America	Mammalia	213	16 320	632	222.84	244.11
	Aves	406	344 145	906	417.14	444.01
S Africa	Mammalia	52	803	39	68.50	84.48
	Aves	355	736 551	827	376.68	445.37
SW Africa	Mammalia	89	1730	63	122.85	132.60
	Aves	55	163	29	68.34	206.31
SE Africa	Mammalia	67	950	48	72.31	91.24
	Aves	302	19 968	118	356.54	378.06

Number of individuals, species and occupied cells are shown along with mean values for the abundance and incidence coverage species richness estimators (ACE, ICE).

These results confirm that there are inequities in available information for birds and mammals (based on GBIF records) in different regions of the world. Bird species richness estimates in some regions (Europe, South Africa and North America) appears to both plateau and converge towards a consistent richness value given the sampling methodologies. In other regions (e.g. south-western Africa, eastern Australia) estimator performance varies more widely and show large percentage increases in richness compared to observed values. Mammal sampling intensity is also variable across the nine regions; some areas appear to converge towards reasonable species richness estimates (e.g. western North America). In other areas, estimators widely vary and do not often reach plateaus (e.g. Africa). In the majority of cases across the nine regions and for the two taxonomic groups, sampling of species using GBIF-mediated records is incomplete; therefore further accumulation of species occurrences from non-networked providers or further field sampling will likely yield significant numbers of new species records.

Three points emerge from this case study. One is the ease with which these global comparisons can be carried out by building tools that partially automate accessing, querying, filtering and storing data from a unified global database of species-occurrence records. The second point is that the GBIF data cache likely does not include the maximum observable number of species, even in well-studied groups like birds and mammals in well-studied areas (e.g. eastern North America). The third point is that the emergence of ‘smart’ web-enabled computer programs to collate data from this global database will accelerate identifying and communicating the strengths and weaknesses in global shared biodiversity data.

### Integrating biodiversity information

Although species-occurrence information forms a core component for biodiversity analyses, by itself it is often not sufficient to answer biodiversity research questions. Useful data about life phase (e.g. juvenile vs. adult), morphology, genetics, physiology or phenology of individual specimens is typically not captured in GBIF-mediated data. However, these data may exist in other large databases that share biological information, such as EMBL and GenBank for genetic resources, or Morphbank (http://www.morphbank.net) for phenotypic resources. Ultimately, users will need to be able to aggregate heterogeneous biological information stored in multiple distributed databases across the world. Indeed, facilitating linkages among such diverse and disparate databases in order to enable queries across all levels of biological organization has been, from its inception, one of GBIF's long-term goals.

In addition, users will need tools for synthesizing information visually and analytically in ways that embrace multiple scales and dimensions of interpretation. A discussion of emerging solutions to the problem of finding (using Life Science Identifiers; http://lsid.sourceforge.net/) and integrating heterogeneous data from multiple distributed databases (using workflow tools like Kepler; http://seek.ecoinformatics.org/Wiki.jsp?page=Kepler) is beyond the scope of this paper. Instead, we describe two studies that use novel visualization approaches and combine multiple sources of biodiversity information – not just point data or abundance distributions, but also information about, for example, evolutionary relationships, functionally important mutations and host–disease interactions. Both of these examples use virtual globes to track the worldwide spread of H5N1 avian influenza.

Butler (2006) provides a novel, visual summary of global H5N1 outbreaks in Google Earth, with symbol colour and size representing differing hosts and number of cases in different geographic areas (http://www.nature.com/nature/multimedia/googleearth/index.html#flu). In a manner similar to KSORD visualizations, this work provides an immediate view of the growing knowledge about locations and number of H5N1 cases. Unlike KSORD, however, the visualization also presents, by representing different host types with different symbols, an additional data dimension that is important for understanding the spread of the disease.

**Figure 5:**
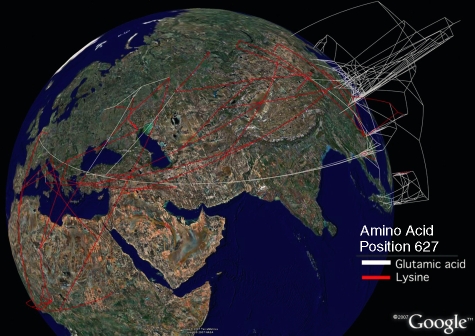
A screenshot from Google Earth showing the spread of H5N1 avian influenza lineages across the Eurasian continent. Here, Janies *et al.* (2007) illustrate the mutation of a key amino acid thought to regulate host switching events in mammals – the lineages coloured red have that mutation while lineages in white do not. Note that the lineages bearing this key mutation arose around the same time that the H5N1 virus spread westward out of eastern Asia.

Janies *et al.* (2007) take Butler's approach in another direction, using Google Earth to represent evolutionary relationships and genomic mutations among H5N1 strains. A phylogenetic hypothesis for H5N1 based on over 300 publicly available full genomes of the virus was constructed and plotted onto Google Earth. With this, the geographic and chronological spread of H5N1 lineages and their associated mutations across the globe (Fig. 5) could be examined. This visualization effectively summarizes a wealth of information about evolution, biogeography, host utilization and even functional genomics of the virus. It can be used by avian influenza specialists and health officials to understand where potentially functionally important mutations are circulating, and to predict where strains might move in the future. Wildlife and food agencies can examine where and when host shifts have occurred during the evolution of the virus. Genomicists can use the maps to determine if mutations known to be functionally important in changing binding properties of surface coat proteins are related to host-switching events. The reason that the mash-up of phylogenetic information onto Google Earth is so rich is because (i) it embraces multiple data dimensions (e.g. geographic, temporal, phylogenetic, genotypic, protein phenotypic, etc.) and (ii) it can be examined at multiple spatial scales, from the global-scale movements of H5N1 out of Australasian flyways westward into Europe (Fig. 5) to local scales, to show, for instance, which lineages reinvaded Hong Kong since the initial spread of the virus.

Visualizations like those in Butler (2006) and Janies *et al.* (2007) not only enable data analysis and communication of results, but also serve to engage the interest of audiences who may otherwise be indifferent to the topics being researched. Visualization tools bridge the gap between researchers and those who most need to be reached with research results, including policy-makers and citizens. Indeed, this may be the area where visualization holds the greatest potential (Kallick-Wakker 1994). The most effective way to harness this potential will be to adopt formats that can be used by the largest number of people. The work done by Janies *et al.* (2007) uses the KML file format of Google Earth. This format is very easy to share with growing millions of Google Earth users. Therefore, the main results from Janies *et al.* (2007) can be opened and manipulated by scientists and non-scientists alike. As additional H5N1 genomes are sequenced and phylogenetic analyses re-run, the visualizations can be updated to show the continued evolution and global spread of the avian influenza virus. Further, the visualizations and analyses can be incorporated into yet other mash-ups that include information that we currently lack, such as bird migratory pathways.

Increasing GBIF data content in a prioritized way and developing visualization tools, analytical methods and web-based workflow mechanisms that meet growing needs for information and knowledge is a fundamentally communal process. Ultimately, such efforts can extend how we communicate biodiversity research ideas and results to a much broader audience than is currently reached using traditional methods. Educating and influencing this broader audience is crucial to our ability to combat the accelerating biodiversity crisis. Whether we do this effectively is dependent on the accuracy of data and efficacy of tools and workflow systems discussed here. More importantly, it depends on collaborative efforts among data providers and users, software and data portal developers, and the biodiversity research community at large.
